# Free adipomuscular diagonal upper gracilis flap as volume replacement for reconstruction of partial breast defect in the medial upper quadrant

**DOI:** 10.1016/j.jpra.2026.05.034

**Published:** 2026-06-02

**Authors:** RJ Erdbrink, J. Nick Brinkman

**Affiliations:** HagaHospital the Hague, Els Borst-Eilersplein 275, 2545 AA The Hague, The Netherlands

**Keywords:** Partial breast reconstruction, Free diagonal upper gracilis flap, DUG, Medial upper quadrant

## Abstract

**Introduction:**

The use of the gracilis flap has been extensively described for whole breast reconstruction, however its application as volume replacement for partial breast defects has only scarcely been described. This short communication covers two patient cases in which a free adipomuscular diagonal upper gracilis (DUG) flap is used as volume replacement for reconstruction of a partial breast defect in the medial upper quadrant of the breast following lumpectomy.

**Case introduction:**

Both patients are healthy middle-aged women who presented themselves with a breast malignancy in the upper medial quadrant of the left breast. Because of the tumor location, their small-to-moderate breast size and nonptotic breasts a free flap reconstruction was selected using a free diagnonal upper gracilis flap.

**Methods/Surgery technique:**

In both patients the flap was harvested conform current techniques.

**Results:**

Both flaps were successful with no evidence of (partial) flap loss. In one patient a wound dehiscence of the donor site was reported, which healed over time by secondary intention. Scar revision surgery was performed to improve the aesthetic appearance of the donor site. Both patients reported high satisfaction with their reconstructions, though the second patient has not yet underwent radiotherapy of the operated breast.

**Conclusion:**

The free adipomuscular gracilis flap represents a valuable option for volume replacement in partial breast reconstruction.

## Introduction

Reconstruction following lumpectomy depends on multiple factors, including breast size, shape, and tumor characteristics. Two principal approaches are commonly employed: volume displacement and volume replacement. While volume displacement is suitable for moderate to large breasts, patients with smaller or non-ptotic breasts often require volume replacement to maintain breast contour and symmetry.^1^

Regional pedicled perforator flaps, such as AICAP, LICAP, and LCAP flaps, are frequently used in oncoplastic surgery. However, reconstruction of defects in the upper medial quadrant remains challenging due to limited local tissue availability and less favorable perforator anatomy.^2^ In such cases, free tissue transfer may provide a reliable alternative.^3^

The gracilis muscle flap, widely used in total breast reconstruction, is characterized by consistent vascular anatomy, low donor site morbidity, and relative technical simplicity.^4^ Its application in partial breast reconstruction has been less frequently reported. This study presents two cases in which a free adipomuscular diagonal upper gracilis (DUG) flap was used for volume replacement following lumpectomy in the upper medial breast quadrant.

## Case introduction

### Patient 1

A 50-year-old woman presented with a 4.2 cm grade 1 hormone receptor-positive invasive carcinoma in the upper medial quadrant of the left breast (cT2N0Mx). Following neoadjuvant endocrine therapy, tumor size decreased to 1.8 cm. She subsequently underwent breast-conserving surgery with lumpectomy and sentinel lymph node biopsy, resulting in a defect measuring approximately 6 × 5 × 2 cm.

Given the tumor location and the patient’s small-to-moderate breast size with minimal ptosis, volume replacement using a free adipomuscular gracilis flap was selected *(see Picture 1).* Reconstruction was performed 17 days after the initial surgery following confirmation of negative margins.

### Patient 2

A 46-year-old woman presented with a 1.5 cm grade 2 hormone receptor-positive invasive carcinoma with associated ductal carcinoma in situ (cT1N0Mx) in the same quadrant. Lumpectomy and sentinel node biopsy resulted in a 4.5 × 3.5 × 3 cm defect. Pathology confirmed complete tumor excision but revealed a macrometastasis in the sentinel node, necessitating adjuvant chemotherapy and endocrine therapy.

Given similar anatomical considerations, reconstruction with a free adipomuscular gracilis flap was performed 14 days postoperatively.

## Surgical technique

In both cases, the gracilis flap was harvested from the left medial thigh using standard techniques. Patients were positioned supine with the thigh slightly abducted and the knee flexed. A handheld Doppler was used to identify perforators. The previous lumpectomy incision was reopened to assess the defect, which was deemed unsuitable for volume displacement. A reduced-size gracilis flap was designed to match the defect volume.

The flap was elevated from anterior to posterior, identifying and preserving the vascular pedicle. Simultaneously, the internal thoracic vessels were exposed for microvascular anastomosis. Due to limited intercostal space, partial resection of costal cartilage was required in both cases. Microvascular end-to-end anastomoses were performed using 9–0 ethilon for the artery and a venous coupler for the vein. The dermis of the skin paddle was completely removed, leaving adipomuscular tissue for volume restoration. Slight overcorrection was applied to compensate for expected muscle atrophy and radiotherapy-related volume loss. Perfusion was confirmed using indocyanine green angiography. No monitoring skin paddle was used. Total operative time ranged from 3 to 4 h.

## Results

Both flaps survived completely without vascular compromise. Patients were discharged on postoperative day two. Patient 1 developed donor site wound dehiscence, likely associated with active smoking. This was managed conservatively with local wound care and negative pressure therapy, followed by successful scar revision at 5.5 months. Donor site wound complications have been reported in gracilis flap surgery and remain an important consideration.^5^ Patient 2 experienced no donor site complications.

Patient 1 received adjuvant radiotherapy (5 × 5.2 Gy) six weeks post-reconstruction. At 8-month follow-up, she reported high satisfaction, with good volume retention, natural consistency, and no evidence of fat necrosis *(see Picture 2)*. The donor site scar was acceptable after revision.

Patient 2, assessed at 2 months postoperatively, was also satisfied, with preserved breast symmetry and no immediate complications, though radiotherapy was ongoing at the time of reporting.

In both cases, satisfactory symmetry was achieved without contralateral procedures.

## Discussion

These cases demonstrate that the free adipomuscular gracilis flap is a viable option for partial breast reconstruction, particularly for defects in anatomically challenging regions such as the upper medial quadrant. The flap provides well-vascularized autologous tissue with favorable handling characteristics and a natural consistency. Its vascular pedicle is reliable and consistent, and donor site morbidity is generally low, with minimal functional impairment and a well-concealed scar.^4^ However, several limitations must be considered. The gracilis flap provides limited volume, which may be insufficient for larger defects and may necessitate adjunctive fat grafting. Furthermore, muscle atrophy and radiotherapy may influence long-term volume retention and aesthetic outcomes.

Donor site complications, including wound dehiscence, seroma, and delayed healing, remain a concern. Smoking is a known risk factor and likely contributed to the complication observed in patient 1.

## Conclusion

**Fig. 1:**
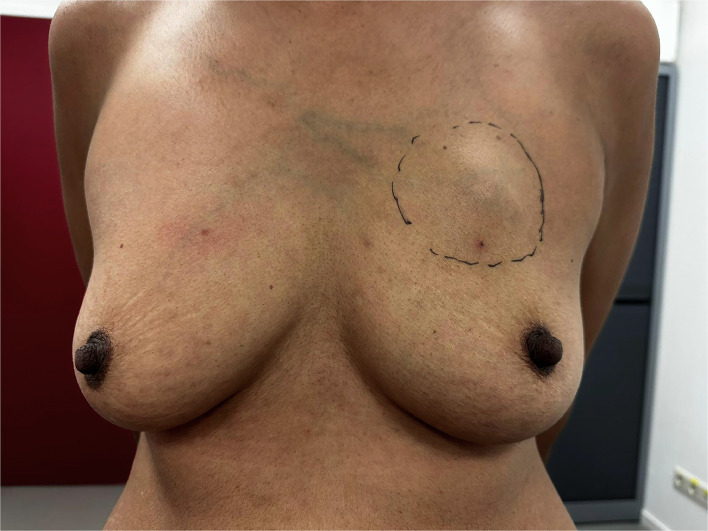
Pre-operative photo of patient 1.

**Fig. 2:**
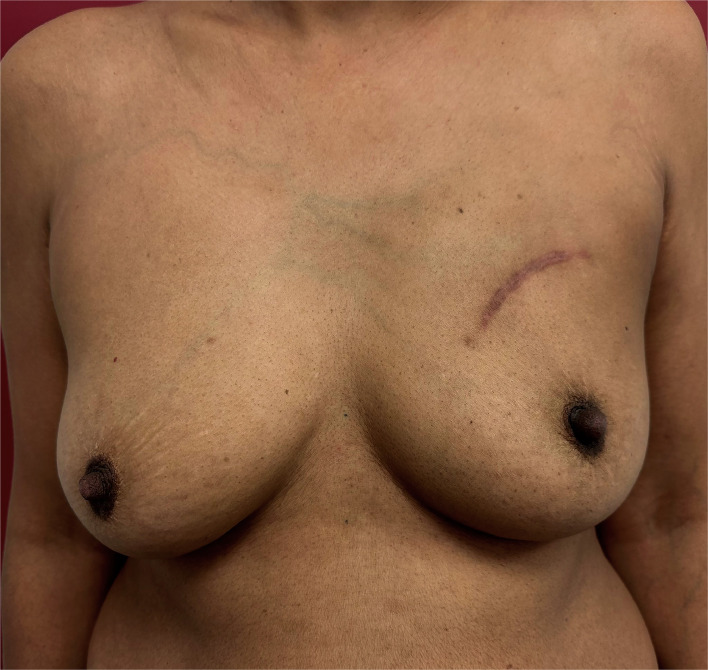
Post-operative (and post-radiotherapy) photo of patient 1.

The free adipomuscular gracilis flap represents a reliable and effective option for volume replacement in partial breast reconstruction following lumpectomy, particularly when local perforator flaps are not feasible. With appropriate patient selection, excellent aesthetic and functional outcomes can be achieved, despite potential donor site morbidity. This technique should be considered a valuable secondary reconstructive option in selected cases (Fig. 1, Fig. 2).

## Funding

None.

## Ethical approval

Research informed consent and photo patient consent was obtained.

## Declaration of generative AI and AI-assisted technologies in the manuscript preparation process

During the preparation of this work the author used OpenEvidence in order to assist with the background literature search. After using this tool, the author reviewed and edited the content as needed and take full responsibility for the content of the published article.

## Declaration of competing interest

None declared.
